# Clinical characteristics, management approaches, and outcomes of patients with heart failure: the Jordan Heart Failure Registry

**DOI:** 10.3389/fcvm.2025.1592002

**Published:** 2025-07-28

**Authors:** Hadi Abu-Hantash, Yousef Khader, Akram AL-Saleh, Hanna Al-Makhamreh, Rasheed Ibdah, Muhannad Ababneh, Asem Nammas, Amr Rasheed, Ahmad Toubasi, Sarah Al-Qalalweh, Mohammad Y. Mahmoud, Farah Albustanji, Yazan Y. Obaid, Hind B. Abu Tawileh, Toqa Awaisheh, Louis Elias Hobeika, Ala Banihamdan, Abdullah Al-Kasasbeh, Sukaina Rawashdeh, Mohammad Abu-hantash, Ali Shakhatreh, Mahmoud Izraiq

**Affiliations:** ^1^ Department of Cardiology, Amman Surgical Hospital, Amman, Jordan; ^2^ Department of Cardiology, Jordan University of Science and Technology, Irbid, Jordan; ^3^ Department of Cardiology, The University of Jordan, Aljubeiha, Jordan; ^4^ Department of Cardiology, King Abdullah University Hospital, Ar Ramtha, Jordan; ^5^ Department of Cardiology, Ibn Al-Haytham Hospital, Amman, Jordan; ^6^ Department of Cardiology, Shmaisani Hospital, Amman, Jordan; ^7^ Department of Cardiology, King Hussein Cancer Center, Amman, Jordan; ^8^ Department of Cardiology, Specialty Hospital, Amman, Jordan

**Keywords:** heart failure, registry, comorbidity, management, outcomes

## Abstract

**Background:**

Heart failure (HF) is a complex clinical syndrome with diverse etiologies and pathophysiological mechanisms. While chronic HF has gained substantial understanding, acute HF remains less elucidated. This study aimed to analyze the data from the Jordanian Heart Failure Registry (JoHFR) to assess the clinical characteristics, management approaches, and outcomes of patients with HF.

**Methods:**

The study included data from 21 healthcare centers from private sector clinics and hospitals and public secondary and tertiary university hospitals across Jordan from July 1, 2021, to February 28, 2023. It included patients above the age of 18 with chronic HF, acute decompensated HF *de novo*, or acute on top of chronic HF. Data encompassed patient demographics, comorbidities, medications, clinical presentation, laboratory findings, echocardiograms, and outcomes.

**Results:**

A total of 2,151 patients were enrolled. More than half (58.0%) of the study cohort were males. The mean age of patients was 66 years, with almost half of the patients (45.5%) aged 70 or older. Chronic HF accounted for 71% of cases, while acute HF constituted 29% of cases. Obesity was identified in 36.6% of the cohort. Hypertension (80.7%) and a history of atherosclerotic cardiovascular disease (ASCVD) (80.6%) were the most prevalent comorbidities. Beta-blockers were the most commonly prescribed medications (67.4%). RAAS inhibitors including ACE inhibitors (18.1%), ARBs (25.2%), and ARNI (10.8%), were also frequently used. Dyspnea (85.9%) was the most common presenting symptom, and elevated B-type natriuretic peptide levels were well utilized and observed in most patients (96.7%). Left ventricular ejection fraction (LVEF) was ≤40% in 58.8%, 41%–49% in 11.4%, and ≥50% in 29.7% of patients, with a mean (SD) of 38.1% (12.7%). The in-hospital mortality rate in our registry stood at 9.6%.

**Conclusion:**

The Jordan Heart Failure Registry (JoHFR) presents the unique characteristics of both chronic and acute HF patients in Jordan and highlights areas for improving patient care and adherence to international guidelines. These findings should guide healthcare policymakers and practitioners in the country to enhance the quality of management of HF patients and implement interventions to reduce the burden of comorbidities accompanying HF.

## 1 Introduction

Heart failure (HF) represents a clinical syndrome characterized by diverse etiologies and pathophysiological mechanisms (1). Consensus guidelines typically employ the term HF to denote patients with well-established chronic HF (CHF), a category that has gained substantial understanding over the past two decades (2). Conversely, acute HF (AHF) remains less comprehensively elucidated. Patients presenting with AHF typically experience a fast onset of the condition, often within the context of pre-existing cardiomyopathy, and their admission to a healthcare facility typically portends an unfavorable prognosis, marked by a high risk of readmission and post-discharge mortality (3).

Patients with HF usually present with a combination of symptoms like shortness of breath, orthopnea, and swelling in the lower extremities, and observable signs such as increased jugular venous pressure and congestion in the lungs. These symptoms and signs often result from structural and/or functional abnormalities in the heart, leading to decreased cardiac output and/or higher pressures within the heart chambers (4). The measurement of left ventricular ejection fraction (LVEF), typically done through Echocardiography and pro-BNP, remains a fundamental aspect of diagnosing and understanding HF. EF and pro-BNP also play a crucial role in predicting outcomes, deciding on the appropriate treatment, and determining the severity of the condition in patients (5).

It is pertinent to underscore that HF stands as one of the leading causes of hospitalization among the adult population, and it constitutes a substantial contributor to healthcare expenditures. It has become a global pandemic, with an increasing prevalence cases of 64 million cases worldwide (3). This rise can be attributed to several factors such as an aging population, improved survival rates after heart attacks, and advancements in treatment options which have led to better survival rates among individuals with HF (6).

Studies involving Middle Eastern populations often encompass diverse ethical and cultural backgrounds that distinguish them from American and European counterparts (7). Consequently, findings from research on HF epidemiology, treatment strategies, and outcomes conducted in other regions may not be readily applicable to the Middle East, particularly Jordan.

The Jordan Heart Failure Registry (JoHFR) represents the first multicenter registry implemented in Jordan and one of the relatively few conducted in the broader Middle East region. The primary objective of this registry is to comprehensively investigate the clinical characteristics, management approaches, and both short- and long-term outcomes of individuals with AHF and CHF. Executed by healthcare cardiology professionals, JoHFR holds a principal mission of bridging knowledge gaps pertaining to HF to enhance the overall well-being and outcomes of patients afflicted by this condition in Jordan. This study aimed to analyze the data from JoHFR to assess the clinical characteristics, management approaches, and outcomes of individuals with AHF and CHF in Jordan.

## 2 Methods

### 2.1 Study design

JoHFR is a computerized registry collecting data about all individuals with acute, pre-existing, or new onset HF presenting to participating centers across Jordan during the enrolment period from July 1, 2021 until February 28, 2023. This included patients with CHF visiting outpatient departments, or acute decompensated HF *de novo* (DNHF) or acute on top of chronic HF (ACHF), admitted to coronary/intensive care unit (CCU/ICU) or wards. Patients initially enrolled as chronic HF who were subsequently admitted with acute decompensation were still analyzed in the chronic HF group for baseline characteristics and long-term outcomes. Their acute events were recorded separately as hospitalizations, without reassigning them to a new cohort. A total of 21 healthcare centers were engaged in this study, comprising a diverse range of facilities, including private sector clinics and hospitals, as well as public and university hospitals. Importantly, participation in the registry did not mandate any modifications to the treatment or hospital care received by patients, and the data input was not contingent upon the utilization of specific therapeutic agents or treatment regimens. Individuals under the age of 18 years were excluded. The 2021 ESC guidelines were used as a reference.

Given the primary focus of this study on quality improvement and assessment, all necessary ethical approvals were obtained, and the survey was approved by each local Institutional Review Board. Prior to data collection, explicit informed consent was obtained from all participating patients.

### 2.2 Data collection

To compile the registry, a dedicated team of cardiologists from each participating institute or clinic undertook the responsibility of generating a comprehensive list of all patients diagnosed with HF. Those cardiologists ensured that more than 95% of consecutively admitted individuals were enrolled in the registry. The patient data collection process involved the initial recording of information on a physical form, which was akin to the format utilized in several other registries. Subsequently, this data was meticulously reviewed to rectify any errors or missing information before being transcribed onto a digital Google Form. This stringent review process ensured the data's accuracy and completeness before submission for final analysis. To prevent the duplication of patient entries, each patient's national identification number was utilized as a unique identifier. The task of data entry was carried out by a collaborative team comprising clinical medical students and residents, cardiologists, and dedicated research assistants.

### 2.3 Data variables

The following dataset was compiled encompassing various patient-related factors: patient demographics, admission date, historical medical information encompassing general and cardiac health (such as hypertension, diabetes, dyslipidemia, arrhythmias, or structural heart disease), HF etiology (including whether it was ischemic or non-ischemic and the specific subtype of non-ischemic etiology), and the number of prior admissions if applicable. Other variables included pre-admission medications, clinical presentation upon admission (including vital signs and NYHA class), comprehensive laboratory investigations such as B-type natriuretic peptide (BNP), electrolyte levels, N-terminal pro-B-type natriuretic peptide (Nt-proBNP), estimated glomerular filtration rate (eGFR), troponin levels, glycated hemoglobin (HbA1c), electrocardiogram (EKG) results, and findings from echocardiograms. Furthermore, information regarding discharge medications, symptom improvement, and post-discharge laboratory values was also gathered. Additionally, for patients admitted with acute decompensated HF, data regarding cardiac procedures and in-hospital outcomes, including mortality, were meticulously documented. Echocardiographic data were also evaluated by experienced cardiology consultants who were part of the daily clinical care team. The 30-day mortality, need for mechanical ventilation, and length of hospital stay were collected for the patients admitted to the hospital.

### 2.4 Statistical analysis

Data were described using percentages for categorical variables and means or medians for continuous variables. Some continuous variables were grouped and described using percentages. Chi-square test was used to compate the demographic, clinical, and relavent chartiristcs of three groups of patients: CHF, ACHF, and DNHF. Data were analyzed using Statistical Package for the Social Sciences (SPSS) version 24 statistical software package.

## 3 Results

### 3.1 Patients’ demographics and baseline characteristics

A total of 2,151 patients were enrolled (1,531 patients with CHF, 387 patients with ACHF, and 233 patients with DNHF). Table 1 presents the demographics and clinical characteristics of study participants categorized by type of heart failure. More than half (58.0%) of the study cohorts were males. Females were less represented in the CHF group (39.4%) compared to the ACHF (46.5%) and DNHF (51.5%) groups. The mean age of patients was 66 years, with almost half of the patients (45.0% of CHF patients, 46.9% of ACHF patients, and 47.5% of DNHF patients) aged 70 or older. Overall, 31.3% of patients were smokers, with no significant difference between the three groups (*P*-value = 0.134). Obesity was prevalent, identified in 36.6% of the cohort. Additionally, 5.3% of patients reported a positive family history of premature atherosclerotic cardiovascular disease (ASCVD), and 80.6% had a personal history of premature ASCVD according to medical records. Atrial fibrillation was present in 30.4% of CHF patients, 39.1% of ACHF patients, and 23.4% of DNHF patients (*P*-value for inter-group differences = 0.002). Additionally, mitral regurgitation (MR) was identified as mild in 51.8% of patients, moderate in 41%, and severe in 7.2%. Almost 69.2% of patients had diabetes (*P*-value < 0.001), 80.7% had hypertension (*P*-value = 0.136), 57.9% had dyslipidemia (*P*-value < 0.001).

**Table 1:** Demographics and baseline characteristics of the study participants.

Variable	Type of heart failure						Total (*N* = 2,151)		*P*-value
	Chronic heart failure (*N* = 1,531)		Acute-on-chronic heart failure (*N* = 387)		*de novo* heart failure (*N* = 233)				
	*n*	%	*n*	%	*n*	%	*N*	%	
Sex									<0.001
Male	922	60.6%	201	53.5%	112	48.5%	1,235	58.0%	
Female	599	39.4%	175	46.5%	119	51.5%	893	42.0%	
Age									0.560
<40	61	4.4%	16	4.3%	10	4.5%	87	4.4%	
40–49	97	7.0%	31	8.4%	14	6.3%	142	7.2%	
50–59	246	17.7%	46	12.5%	35	15.8%	327	16.5%	
60–69	362	26.0%	103	27.9%	57	25.8%	522	26.3%	
≥70	627	45.0%	173	46.9%	105	47.5%	905	45.6%	
Smoking	458	32.2%	117	31.5%	52	25.2%	627	31.3%	0.134
Obesity									0.848
Normal	234	27.0%	58	26.0%	37	25.0%	329	26.6%	
Overweight	324	37.4%	77	34.5%	56	37.8%	457	36.9%	
Obesity	309	35.6%	88		55	37.2%	452	36.5%	
Family history of premature ASCVD	90	6.3%	5	1.3%	12	5.8%	107	5.3%	0.001
Diabetes mellitus	956	67.0%	290	77.7%	142	68.6%	1,388	69.2%	<0.001
Hypertension	1,136	79.8%	313	84.4%	166	80.6%	1,615	80.7%	0.136
Dyslipidemia	812	57.0%	244	65.8%	102	49.5%	1,158	57.9%	<0.001
History of ASCVD	943	81.6%	227	76.4%	111	81.0%	1,281	80.6%	0.135
Atrial fibrillation	352	30.4%	116	39.1%	32	23.4%	500	31.4%	0.002
History of structural heart disease	79	6.8%	13	4.4%	7	5.1%	99	6.2%	0.251

ASCVD, atherosclerotic cardiovascular disease.

### 3.2 Medications

The management of HF in this cohort involved a diverse array of medications, detailed in Table 2. Among the key medications, beta-blockers were the most common and used by 67.4% of patients (66.3% of CHF patients, 77.5% of ACHF patients, and 56.8% of DNHF patients). RAAS inhibitors—encompassing ACE inhibitors (18.1%), ARBs (25.2%), and ARNI (10.8%)—were frequently used. Aldosterone antagonist usage was 20.3% in CHF, 27.9% in ACHF, and 10.2% in DNHF, showing a significant difference (*P*-value < 0.001). SGLT2 inhibitors were used by 9% of patients. Loop diuretics were used by 51.6% of patients. Additionally, statins (33.2%), aspirin (59.8%), and metformin (45.3%) were commonly used, whereas digoxin was used by a mere 5.4% of patients. The combination of RAAS inhibitors and beta-blockers was used by 41% of patients. The usage of antiarrhythmic medications was low across all groups, with an overall usage of 2.2% (*P*-value = 0.182). Calcium channel blocker usage was 17.9% in CHF, 22.5% in ACHF, and 22.3% in DNHF, with no significant difference (*P*-value = 0.062).

**Table 2:** Home medications among patients with heart failure.

Medication	Chronic heart failure (*N* = 1,531)		Acute-on-chronic heart failure (*N* = 387)		*de novo* heart failure (*N* = 233)		Total (*N* = 2,151)		*P*-value
	*n*	%	*n*	%	*n*	%	*N*	%	
RAAS Inhibitor	756	52.7%	184	48.8%	81	34.8%	1,021	50.6%	0.001
Aldosterone Antagonist	292	20.3%	105	27.9%	21	10.2%	418	20.7%	<0.001
Beta Blocker	951	66.3%	292	77.5%	117	56.8%	1,360	67.4%	<0.001
SGLT2 inhibitors	64	10.1%	15	7.1%	6	5.9%	85	9.0%	0.220
ACE inhibitors	274	19.1%	65	17.2%	26	12.6%	365	18.1%	0.070
ARB	357	24.9%	99	26.3%	52	25.2%	508	25.2%	0.859
ARNI	175	12.2%	34	9.0%	8	3.9%	217	10.8%	0.001
Antiarrhythmic	36	2.5%	8	2.1%	1	0.5%	45	2.2%	0.182
Calcium Channel Blocker	257	17.9%	85	22.5%	46	22.3%	388	19.2%	0.062
Loop Diuretics	689	48.0%	279	74.0%	73	35.4%	1,041	51.6%	<0.001
Thiazide Diuretics	159	11.1%	31	8.2%	26	12.6%	216	10.7%	0.180
Anticoagulant	269	17.6%	75	19.4%	48	20.6%	392	18.2%	0.434
Statin	478	33.3%	136	36.1%	56	27.2%	670	33.2%	0.092
Other lipid lowering agents	277	19.3%	89	23.6%	34	16.5%	400	19.8%	0.079
Aspirin	841	58.6%	253	67.1%	113	54.9%	1,207	59.8%	0.003
Other antiplatelet	247	17.2%	76	20.2%	18	8.7%	341	16.9%	0.002
Digoxin	76	5.3%	32	8.5%	1	0.5%	109	5.4%	<0.001
Nitrate	130	9.1%	41	10.9%	10	4.9%	181	9.0%	0.051
Metformin	288	45.5%	86	40.8%	54	53.5%	428	45.3%	0.106
DPP-4 inhibitors	90	14.2%	44	20.9%	13	12.9%	147	15.6%	0.052
Insulin	333	52.6%	103	48.8%	52	51.5%	488	51.6%	0.634
Sulfonylurea	88	13.9%	39	18.5%	14	13.9%	141	14.9%	0.257

RAAS, renin-angiotensin-aldosterone system; SGLT2, sodium-glucose co-transporter-2; ACE, angiotensin-converting enzyme; ARB, angiotensin 2 receptor blocker; ARNI, angiotensin receptor/neprilysin inhibitor; DPP-4, dipeptidyl peptidase 4.

### 3.3 Clinical and laboratory characteristics at presentation

Upon presentation, dyspnea emerged as the most prevalent symptom (85.9%; 84.2% of CHF patients, 89.5% of ACHF patients, and 89.8% of DNHF patients (*P*-value = 0.007), followed by orthopnea (39.5%;31.3% of CHF patients, 60.9% of ACHF patients, and 51.1% of DNHF patients) and chest pain (32.8%). Fatigue was experienced by 28.5% of patients (*P*-value = 0.004), palpitations by 9.6% (*P*-value = 0.016), and paroxysmal nocturnal dyspnea (PND) by 30.6% (*P*-value < 0.001).

Elevated systolic blood pressure (SBP ≥ 140 mmHg) was observed in 6.7% of CHF patients, 11.6% of ACHF patients, and 10.3% of DNHF patients (*P*-value < 0.001). Elevated diastolic blood pressure (DBP ≥ 90 mmHg) was noted in 5.1% of CHF patients, 7.4% of ACHF patients, and 9.8% of DNHF patients (*P*-value < 0.001). Elevated cholesterol levels (>200 mg/dl) were found in 14.2% of patients (*P*-value = 0.007). Low HDL levels (≤45 mg/dl) were reported in 72.2% of patients (*P*-value < 0.001), elevated LDL levels (≥130 mg/dl) in 12.5% (*P*-value < 0.001), and elevated triglycerides (≥150 mg/dl) in 36.8% (*P*-value < 0.001).

Laboratory investigations revealed significantly elevated levels of B-type natriuretic peptide (BNP) in 96.7% of patients (95.0% of CHF patients, 99.1% of ACHF patients, and 98.8% of DNHF patients) and N-terminal proBNP (NT-proBNP) in 90.9% of patients (88.6% of CHF patients, 92.7% of ACHF patients, and 97.8% of DNHF patients). Some patients experienced electrolyte imbalances, with 30% having sodium levels below 136 mmol/L and 13% having potassium levels above 5 mmol/L. Moreover, 62.2% had hemoglobin levels below recommended thresholds, but a majority (83.5%) had levels above 10 g/dl. Approximately 42.6% displayed impaired kidney function (eGFR <60 ml/min/1.73 m²), while elevated creatinine levels (>115 µmol/L) were noted in 42.5% of patients. Furthermore, 64.2% exhibited elevated hemoglobin A1c (HbA1c) levels (>6%). LVEF was ≤40% in 58.8%, 41%–49% in 11.4%, and ≥50% in 29.7% of patients, with a mean (SD) of 38.1% (12.7%) (Table 3). The in-hospital mortality rate in our registry stood at 9.6%.

**Table 3:** Clinical characteristics of patients with heart failure at presentation.

Symptoms	Chronic heart failure (*N* = 1,531)		Acute-on-Chronic heart failure (*N* = 387)		*de novo* heart failure (*N* = 233)		Total (*N* = 2,151)		*P*-value
	*n*	%	*n*	%	*N*	%			
Dyspnea	1,079	84.2%	334	89.5%	202	89.8%	1,615	85.9%	0.007
Fatigue	388	30.3%	80	21.4%	67	29.8%	535	28.5%	0.004
Chest pain	396	30.9%	138	37.0%	83	36.9%	617	32.8%	0.034
Palpitation	140	10.9%	23	6.2%	18	8.0%	181	9.6%	0.016
Orthopnea	401	31.3%	227	60.9%	115	51.1%	743	39.5%	<0.001
PND	298	23.3%	185	49.6%	92	40.9%	575	30.6%	<0.001
Vital signs									
SBP ≥ 140	66	6.7%	26	11.6%	14	10.3%	106	7.9%	<0.001
DBP ≥ 90	59	5.1%	20	7.4%	16	9.8%	95	6.0%	<0.001
Cholesterol >200 mg/dl	87	15.5%	8	5.9%	18	18.4%	113	14.2%	0.007
HDL ≤45 mg/dl	401	72.4%	99	72.3%	70	71.4%	570	72.2%	<0.001
LDL ≥130 mg/dl	72	12.7%	11	8.0%	18	17.5%	101	12.5%	<0.001
TG ≥150 mg/dl	214	37.9%	42	30.7%	37	38.5%	293	36.8%	<0.001
High BNP	396	95.0%	227	99.1%	83	98.8%	706	96.7%	<0.001
High NT-proBNP	178	88.6%	76	92.7%	45	97.8%	299	90.9%	<0.001
Sodium (mmol/L)									0.383
<136	412	28.9%	128	34.0%	67	30.3%	607	30.0%	
136–145	959	67.3%	237	63.0%	145	65.6%	1,341	66.3%	
>145	54	3.8%	11	2.9%	9	4.1%	74	3.7%	
Potassium (mmol/L)									0.332
<3.5	69	4.9%	24	6.4%	15	6.8%	108	5.4%	
3.5–5	1,176	82.8%	297	79.2%	174	78.4%	1,647	81.6%	
>5	176	12.4%	54	14.4%	33	14.9%	263	13.0%	
Hb (g/dl): <13 (males)/ <12 (females)	847	61.6%	212	65.2%	126	61.2%	1,185	62.2%	<0.001
eGFR <60 ml/min/1.73 m^2^	202	42.0%	35	54.7%	40	38.1%	277	42.6%	<0.001
BUN >20 mg/dl	921	73.6%	292	80.7%	168	81.6%	1,381	75.9%	<0.001
HbA1c > 6%									0.266
<5.7	150	24.4%	35	21.6%	25	21.9%	210	23.6%	
5.7–6.4	153	24.9%	31	19.1%	23	20.2%	207	23.2%	
≥6.5	312	50.7%	96	59.3%	66	57.9%	474	53.2%	
Cr >115 µmol/L	549	40.4%	198	52.7%	82	38.1%	829	42.5%	<0.001

PND, paroxysmal nocturnal dyspnea; SBP, systolic blood pressure; DBP, diastolic blood pressure; HDL, high density lipids; LDL, low density lipids; TG, triglycerides; BNP, brain natriuretic peptide; NT-proBNP, N-terminal pro–B-type natriuretic peptide; Hb, hemoglobin; eGFR, estimated glomerular filtration rate; BUN, blood urea nitrogen; HbA1c, glycated hemoglobin; EF, ejection fraction; Cr, creatinine.

## 4 Discussion

This is the first national large-scale multi-center registry for patients with HF in Jordan. This study included multiple centers representing the diversity of Jordanian HF patients, spanning both government and private sectors and university and non-university hospitals, ensuring a representative dataset.

Our study revealed that the mean age of HF patients in Jordan is approximately 66 years, slightly higher than that reported in the Saudi HF registry (HEARTS) at 60.6 years (8). In comparison, the ESC-HF pilot survey documented a mean age of 70 years for patients with acute HF and 67 years for chronic HF, while the ADHERE registry reported a comparable mean age of 72.4 years (8–10). The consistency of our findings with previous registries and the evidence that HF presents earlier in Eastern countries compared to Western countries highlights the importance of understanding the unique epidemiological characteristics of HF in our region. Reported data highlighting an average age at least 10 years younger than Western counterparts is alarming (10). This trend in Arab countries, including Jordan, can be attributed to multiple factors. A key contributor is the higher prevalence of atherosclerotic cardiovascular disease (ASCVD) in the region, which tends to affect individuals at a younger age, leading to earlier onset of HF. In our registry, a history of ASCVD was found in 80.6% of patients. This contrasts with the Egyptian registry, where 68.1% of HF patients had ischemic heart disease (IHD) as the primary etiology of HF (1).

Additionally, in the Saudi HEARTS registry, ischemia was associated with HF in 50% of acute HF patients and 41.8% of chronic HF patients (11). By contrast, the ESC-HF pilot survey indicated an ischemic etiology in 50.7% of acute HF patients and 40.4% of chronic HF patients, while the ADHERE registry reported a percentage of 57%. Notably, a significant prevalence of risk factors associated with ischemic heart disease (IHD) in Arab countries is a key contributing factor. The prevalence of diabetes mellitus (DM) in our study population was significantly elevated, with rates of 69.2% among patients with HF, exceeding the rates observed in other registries, including 60.7% in the Saudi HEARTS registry for acute HF and 53% for chronic HF, and 45.4% in the Egyptian registry (1, 12). In contrast, the ESC-HF-pilot survey reported lower DM rates of 35% in AHF and 29% in CHF (9). Similarly, we observed obesity, defined as a body mass index (BMI) of 30 or higher, in 36.5% of HF patients, which, while lower than the Egyptian registry's rate of 46.9%, still indicates a significant prevalence (1). A substantial prevalence of hypertension (HTN) was observed, with 80.7% of patients having HTN. This rate significantly exceeds the 43.5% reported in the Egyptian registry, and it is closer but still higher than the 70% prevalence among acute HF patients reported in the HEARTS Saudi registry (1, 11, 12). It is also higher than the rates reported in the ADHERE registry and the ESC-HF pilot survey (8, 9).

Within this registry, 31.3% of HF patients were identified as smokers, a figure lower than the reported 41% among current conventional tobacco smokers in the Jordan National Stepwise Survey (STEPs) for Noncommunicable Diseases Risk Factors in 2019 (13). It is worth noting that Jordan stands as the foremost country globally for smoking (13). Considering smoking cessation's pivotal role in managing HF patients, there is a pressing need for further actions and interventions to promote smoking cessation and diminish tobacco use among the public.

Atrial Fibrillation was observed in 31.4% of the patients in this study, which is a higher prevalence compared to the rates reported by the Saudi HEARTS registry and the Egyptian cohort (7, 8). It is comparable to the findings in the ADHERE registry but slightly lower than the rate reported by the ESC-HF pilot survey (9, 10). Notably, 70.3% of our patients had an ejection fraction (EF) of less than 50%. The coexistence of atrial fibrillation and left ventricular dysfunction is associated with worse outcomes (14). This underscores the need for randomized controlled trials (RCTs) to establish guidelines for managing atrial fibrillation in the context of HF, addressing aspects such as rate vs. rhythm control and evaluating medical vs. non-medical treatment options. It is worth noting that this prevalence rate is similarly high to rates reported by other registries.

The in-hospital mortality rate in our registry stood at 9.6%, which exceeded the mortality rates reported in other registries, including the Saudi HEARTS registry, the Egyptian cohort, ADHERE, ATTEND, the EHFS II and the OPTIMIZE-HF registries (7, 8, 10, 15–17). Several factors may contribute to this difference. It is important to acknowledge that various registries have implemented different exclusion criteria. For instance, the ATTEND registry excluded patients with ACS, which could influence the reported mortality rates (15). As we did not perform a multivariate analysis to identify independent predictors of mortality, we are cautious in attributing causality to any single factor. However, the high-risk profile of the patients included in our cohort, along with delays in presentation and admission, likely played an important role.

The rate of use of evidence-based therapies in this registry is as follows: Beta-blockers, ACE-I, ARBs, aldosterone antagonists, and ARNI were used in 67.4%, 18.1%, 25.2%, 20.7%, and 10.8% of patients, respectively. These utilization rates are not satisfactory when compared to other registries, which have reported much higher rates, including the Saudi HEARTS registry and the Egyptian cohort (7, 8), especially considering that most of the patients were treated in university hospitals, which should ideally demonstrate the highest rate of evidence-based medicine utilization and guideline-directed practice in the country. The cost of these medications could partially explain their underutilization, as Jordan's national insurance covers many of these drugs. Furthermore, the presence of contraindications in a subset of patients, such as those with chronic kidney disease, may also contribute to the underutilization of these drugs. Nevertheless, these numbers are unsatisfactory and demand intervention to enhance the utilization of evidence-based therapies and optimize the care provided to HF patients in Jordan. Of note that the systolic blood pressure, eGFR, and potassium levels should have encouraged our cardiologists to use the four pillars of medications recommended by ESC and ACC/AHA guidelines.

Our registry's median length of hospital stay is 6.2 days, comparable to the results reported in the Egyptian cohort, ADHERE, and EHFS II, ranging from 4 to 9 days (8, 10, 16) and 8.2 days reported in ESC Atlas registry (9).

The underutilization of implantable cardioverter-defibrillators (ICD) and CRT (cardiac resynchronization therapy) devices in hospitalized patients in Jordan at a rate of 4% is a matter of concern but probably reflects financial constraints. These rates are unsatisfactory, especially considering that most of our patients have left ventricular dysfunction. Also, these rates are notably as low as those reported in counterparts in other Arab countries. For instance, Egypt reported a percentage of 1.5% among hospitalized patients, while the Saudi HEARTS registry reported rates of 29% for ICDs and 8% for CRT devices (7, 8).

However, several limitations should be acknowledged. Like many other registries, hospital participation in our study was voluntary. This means that our findings might not accurately reflect clinical practices across all hospitals in the country. Additionally, hospitals that chose to be part of the registry could be more enthusiastic about following guidelines and improving the quality of care. The observational nature of the study design introduces an inherent selection bias. The relatively small sample size drawn from a limited number of tertiary care hospitals limits the generalizability of our findings to the broader healthcare landscape in our nation. Despite these limitations, this report aims to outline the study's overall design and provide preliminary results from the pilot phase.

In conclusion, JoHFR stands out as the inaugural multicentre HF registry and quality enhancement initiative in both the Hashemite Kingdom of Jordan and the Arab population as a whole. Our findings have illuminated several noteworthy trends. Firstly, our HF patients tend to present at a relatively young age. Secondly, they exhibit markedly higher rates of diabetes mellitus when compared to patients in more developed nations. Thirdly, a significant majority of these patients manifest left ventricular systolic dysfunction, primarily attributed to ischemic causes. In addition, we presented a higher mortality rate than described in previous registries. The preliminary results from our study highlight potential areas for enhancing patient care, mainly utilizing guideline-directed medical therapy, notably the four pillars of medications and the consideration of implanting ICD/CRT devices in individuals with HF with reduced ejection fraction when indicated. These preliminary results should guide governments and health ministries in Jordan and surrounding countries to enhance the quality of the patients care in addition to starting initiatives that reduce the burden of comorbidities among HF patients and improve adherence to the management international guidelines.

## Data Availability

The raw data supporting the conclusions of this article will be made available by the authors, without undue reservation.
